# Defensive behavior of the invasive alien hornet, *Vespa velutina*, against color, hair and auditory stimuli of potential aggressors

**DOI:** 10.7717/peerj.11249

**Published:** 2021-04-06

**Authors:** Moon Bo Choi, Eui Jeong Hong, Ohseok Kwon

**Affiliations:** 1 School of Applied Biosciences, College of Agriculture and Life Sciences, Kyungpook National University, Daegu, Republic of Korea; 2 Institute of Agricultural Science and Technology, Kyungpook National University, Daegu, Republic of Korea; 3 Team of National Ecosystem Survey, National Institute of Ecology, Seocheon-gun, Republic of Korea

**Keywords:** Colour, Hair, Natural enemy, Noise, *Vespa velutina*

## Abstract

**Background:**

During recent years, invasion of the yellow-legged hornet (*Vespa velutina*) has occurred in Europe, Korea and Japan, and stinging accidents often occur as some *V. velutina* nests are in places where humans can reach them. Misleading information regarding precautionary measures for mitigating wasp attacks has only exacerbated the situation. In this study, we sought to identify appropriate countermeasures by analyzing wasp defensive behavior, with a focus on color, hair and auditory stimuli.

**Methods:**

Defensive behavior was analyzed using video recordings by creating an experimental frame to attach experimental bundles to nine *V. velutina* nests in Daegu and Gyeongbuk, South Korea. For the color experiment, eight-color and single-color tests were conducted with bundles of eight colors (black, brown, yellow, green, orange, gray, red and white), and the difference in defensive behavior was tested between black hair/hairless and green hair/black hairless configurations.

**Results:**

When presented simultaneously with bundles of eight different colors, *V. velutina* showed the greatest and the longest defensive behavior against the black bundle, followed by brown. A similar response was observed in single-color tests. Furthermore, there was no significant difference in the defensive behavior against black hair and black hairless, but the duration of defensive behavior was longer for black hair. A comparison between green hair and black hairless stimuli indicated that wasps are more sensitive to color than to hair texture. *Vespa velutina* showed no discernible responses when exposed to selected auditory stimuli (human conversation and loud music). Dark colors and dark hair are characteristic features of potential predators, to which wasps are evolutionarily predisposed, and are accordingly likely to provoke strong defensive responses. The results of this study provide scientifically credible information that can be used to base appropriate precautionary measures against wasp attacks.

## Introduction

Social Hymenoptera, such as hornets, paper wasps, yellowjackets and honeybees, are potentially dangerous insects for people because they sting. Although honeybee stings may be more common than wasp stings (at least in Europe) because honeybees interact more closely with humans, it is the perception of humans that wasps are much more dangerous (Sumner, Law & Cini, 2018). In particular, *Vespa* species of Vespidae are large in size, have a large amount of venom, and are highly aggressive, causing serious injuries and deaths if there is unexpected contact with the nest (Matsuura & Yamane, 1990; Archer, 2012).

In the Southeast Palaearctic regions, where their density is high, tens of thousands of stings occur annually (Xuan et al., 2010; Xie et al., 2013; Tan, Van Achterberg & Chen, 2015; Choi, Kim & Kwon, 2019; Gunasekara et al., 2019). In Korea, China and Japan, in particular, as the number of *Vespa* species in densely populated urban areas has increased, there has been a rapid escalation in the stinging incidents attributable to these wasps (Choi, Kim & Lee, 2012; Choi, Martin & Lee, 2012; Tan, Van Achterberg & Chen, 2015; Azmy, Hosaka & Numata, 2016; Choi, Kim & Kwon, 2019). In contrast, the two species (*V. crabro* and *V. velutina*) in Europe do not pose a significant threat due to the low chance of human contact due to the habitat and characteristics of the nest (De Haro et al., 2010).

In social wasps, natural selection acts via kin selection, whereby individuals in groups work cooperatively to promote the interests of the entire group (Queller & Strassmann, 1998), including the deployment of various defensive strategies designed to protect colonies from invaders (Jeanne, 1981; Judd, 1998, 2000; Bruschini, Cervo & Turillazzi, 2008; Monceau, Bonnard & Thiery, 2013; Cheng et al., 2017). In the presence of an intruder, the older workers generally act as the first line of defense (Jeanne, Williams & Yandell, 1992; Judd, 2000; Togni & Giannotti, 2010; Monceau, Bonnard & Thiery, 2013), initially vibrating their abdomens to generate a rustling sound or vibrating their wings as a warning signal, indicating the intent to fly. If this deterrence proves insufficient, some workers will sting in swarms, spraying alarm pheromones and venom (Jeanne, 1981; Jeanne & Keeping, 1995; Judd, 1998). These defensive behaviors vary in intensity depending on the external status of the predator or the level of stimulation.

In general, predators of social wasps in the forest are birds such as buzzards and bee-eaters, or mammals such as bears and martens (Zielinski, Spencer & Barrett, 1983; Choi, Woo & Choi, 2015; Seryodkin, 2016; Macià et al., 2019). Most of the predators recognized by wasps in cities will be humans (Matsuura & Yamane, 1990). Consequently, as the area of human activity expands, there is an increased possibility of wasp-human interactions, and thus a heightened risk of wasp-inflicted injury.

During recent years, the yellow-legged or Asian hornet (*Vespa velutina* Lepeletier, 1836) has invaded areas in Europe (Husemann et al., 2020; Rome & Villemant, 2020), Korea (Kim, Choi & Moon, 2006) and Japan (Sakai & Takahashi, 2014), with diverse effects in the areas of economy, public health and ecology (Jung, Kang & Kim, 2007; Choi, Martin & Lee, 2012; Monceau, Bonnard & Thiery, 2014; Cini et al., 2018; Choi, Kim & Kwon, 2019; Yamasaki et al., 2019). In particular, *V. velutina* has had a serious effect on the beekeeping industry in the invaded countries, on account of its predation on foraging honeybees (Jung, 2012; Choi & Kwon, 2015; Requier et al., 2019). In addition, as *V. velutina* has spread into forest and urban areas, accidents often occur in which people are stung and injured (Choi, Martin & Lee, 2012; Fournier et al., 2017; Kennedy et al., 2018; Choi, Kim & Kwon, 2019; Do et al., 2019). Indeed, in Europe, *V. velutina* is considered to have the potential to become the most malignant, invasive species to date (European Commission, 2016; Turchi & Derijard, 2017). In Korea, it was designated as an “Ecological Disturbance Organism” in July 2019, according to the Ministry of Environment Notice, 2019-185 (https://www.law.go.kr/LSW/conAdmrulByLsPop.do?&lsiSeq=212233&joNo=0002&joBrNo=0&datClsCd=010102&dguBun=DEG&lnkText=%25ED%2599%2598%25EA%25B2%25BD%25EB%25B6%2580%25EC%259E%25A5%25EA%25B4%2580%25EC%259D%25B4%2520%25EC%25A7%2580%25EC%25A0%2595%25E3%2586%258D%25EA%25B3%25A0%25EC%258B%259C%25ED%2595%2598%25EB%258A%2594&admRulPttninfSeq=6116, access date 21 October 2020), and consequently, the government has passed legislation relating to the control and management of this species.

*Vespa velutina* also acts defensively against stimuli from external intruders. However, *V. velutina*, like *V. crabro* in Europe and other *Vespa* species in Asia, do not often build their nests in low locations, such as underground or in shrubs, and 70% hang over 10 m high (Villemant et al., 2011; Bessa et al., 2016). Therefore, the public health threat caused by *V. velutina* is not considered significant (De Haro et al., 2010). However, the remaining 30% build nests at less than 10 m, and most of them are built on artificial structures, shrubs and underground (Villemant et al., 2011; Bessa et al., 2016). About 10% build in places that are about the height of a person (~2 m or less) (Bessa et al., 2016). Thus, although the percentage of low-height nests is low, the number of nests per region, city, or country corresponds to thousands of nests per year. Although not more than other wasps, stinging accidents by *V. velutina* do occur often, and as they continue to spread (Villemant et al., 2011; Lioy et al., 2019; Laurino et al., 2020), there is a strong possibility that stinging accidents will also increase.

Therefore, we investigated whether the invasive hornet, *V. velutina*, which is continuously spreading, shows differences in defensive behavior depending on the appearance of predators, such as color, hair and auditory stimuli. This can elucidate how social wasps evolved behaviorally against their natural enemies. By providing accurate scientific information about stinging incidents, mitigation strategies can be formulated.

## Materials and Methods

### Experimental nests

This experiment was conducted on a total of nine *V. velutina* nests (Table 1). Among them, four nests were found on the campus of Kyungpook National University in Daegu, which is an urban area, and the remaining five nests were identified by wasp hunters working in rural areas such as Gunwi, Andong and Hyeonpung in South Korea. The experiment was conducted from late July to early September 2020 (Table 1). Experiments were conducted by finding nests at a 5 m height that could threaten or directly attack humans, or by moving nests from a high position to a height suitable for the experiment. Since there was a risk of passers-by being stung while conducting the experiment, a time period with little traffic or blocked traffic was chosen, or the nest was moved to an experimental area. Nests 2, 3 and 6 were located on the campus of Kyungpook National University, and these are located in the corners of the green area of the campus, where people rarely crowd. Nevertheless, in order to prevent accidents, the experiment was conducted for 5–6 h from 10:00 on Sunday during the summer vacation period in July–August when less traffic was anticipated. In addition, researchers were placed on every street 20 m away from the experimental site to divert possible passers-by. Of the nests found in rural areas, nests 1 and 5 were tested under the eaves of a house with the permission of the owner of the house, and nests 4 and 7 in the trees and shrubs by the side of a rural road were blocked for 20 m by the researchers, even though it was a low traffic road. The experiment was conducted after controlling the road for 6 h.

**Table 1:** Characteristics and information of *V. velutina* nests for experimentation.

Nest no.	Region/coordinates	Habitat type	Nest sites	Height (m)	Nest size (cm) (length × wide × height)	Experiment date	Comb number	Number of workers^1^
1	Geummae-ri, Gunwi, GB N36°08′13.72″ E128°36′39.53″	Rural	Eaves	3	21 × 16 × 19	24 July	3	108
2	KNU, Daegu N35°53′27.78″ E128°36′33.36″	Urban	Window frame	3	16 × 14 × 16	26 July	2	69
3	KNU, Daegu N35°53′18.93″ E128°36′15.99″	Urban	Tree	5	23 × 20 × 27	16 August	3	188
4	Gisa-ri, Andong, GB N36°38′17.83″ E128°52′28.86″	Rural	Tree	4	26 × 23 × 32	20 August	3	233
5	TaeJang-ri Andong, GB N36°38′44.30″ E128°39′35.03″	Rural	Eaves	4	25 × 24 × 20	21 August	3	167
6	KNU, Daegu N35°53′37.33″ E128°36′44.42″	Urban	Ceiling	5	27 × 25 × 22	23 August	3	254
7	Hyeonpung, Daegu N35°41′9.79″ E128°24′54.97″	Rural	Shrub	1	20 × 18 × 20	28 August	3	97
8^2^	KNU, Daegu N35°53′25.74″ E128°36′28.26″ (moved to Gayasan National Park) N35°47′06.71″ E128°03′37.42″	Urban (moved to forest)	Treetop	13 (3)	35 × 24 × 37	31 August	5	489
9^2^	Janggi-ri, Gunwi, GB N36°07′18.16″ E128°34′53.62″ (moved to Mt. Palgongsan) N35°59′40.66″ E128°40′51.09″	Rural (moved to forest)	Treetop	15 (3)	37 × 26 × 44	11 September	6	845

**Notes:**

1 Counting only the individuals left in the nest after the experiment is over.

2 Moved the nest to the forest area and conduct the experiment after re-installation.

GB = Gyeongbuk province; KNU = Kyungpook National University campus.

Nests 8 and 9 were found at the top of a tree (>15 m) on August 24 and September 4, respectively. When approaching the nest with a crane, the workers were observed to show normal behavior with respect to feeding activities, nest construction and defense. I thus waited approximately 10 min for the workers to become less active, and then blocked the entrance to the nests and collected each in a steel cage (65 × 65 × 65 cm). The experiment was continued by moving the nests to controlled areas in Gayasan National Park and Mt. Palgongsan where there is no human access and experiments are possible. These two nests were securely tied to a tree branch 3–4 m high with a rope, and the entrance was carefully opened 1 h after the wasps calmed down. After that, the nests were left in the forest for a week so that normal activities, such as feeding and nest construction, could be carried out to adapt to the new environment. After confirming that the workers were working normally, this experiment was conducted. All the researchers who participated in this experiment wore white protective clothing and tested safe from worker attacks. Of the nests used in the experiment (removed and analyzed after the experiment was over), nests 1–7 were highly likely to be primary nests because they were collected in August at relatively low heights. They were small to medium-sized nests, with 70–250 workers in two to three combs. Nests 8 and 9 at the top of the tree were not at their peak yet but were large second nests with 450–850 workers in five to six combs.

In order to induce the defensive behavior of workers in the experimental nests, the behavior of the workers was recorded after hitting the nest five times with the same intensity at 1 s intervals with a stick, 30 cm away from the nests. The temperature on experimental days was between 28 and 33 degrees, and procedures were conducted on sunny days to avoid climatic interference such as that from rain or typhoons.

### Experimental target and video recording

**Figure 1:**
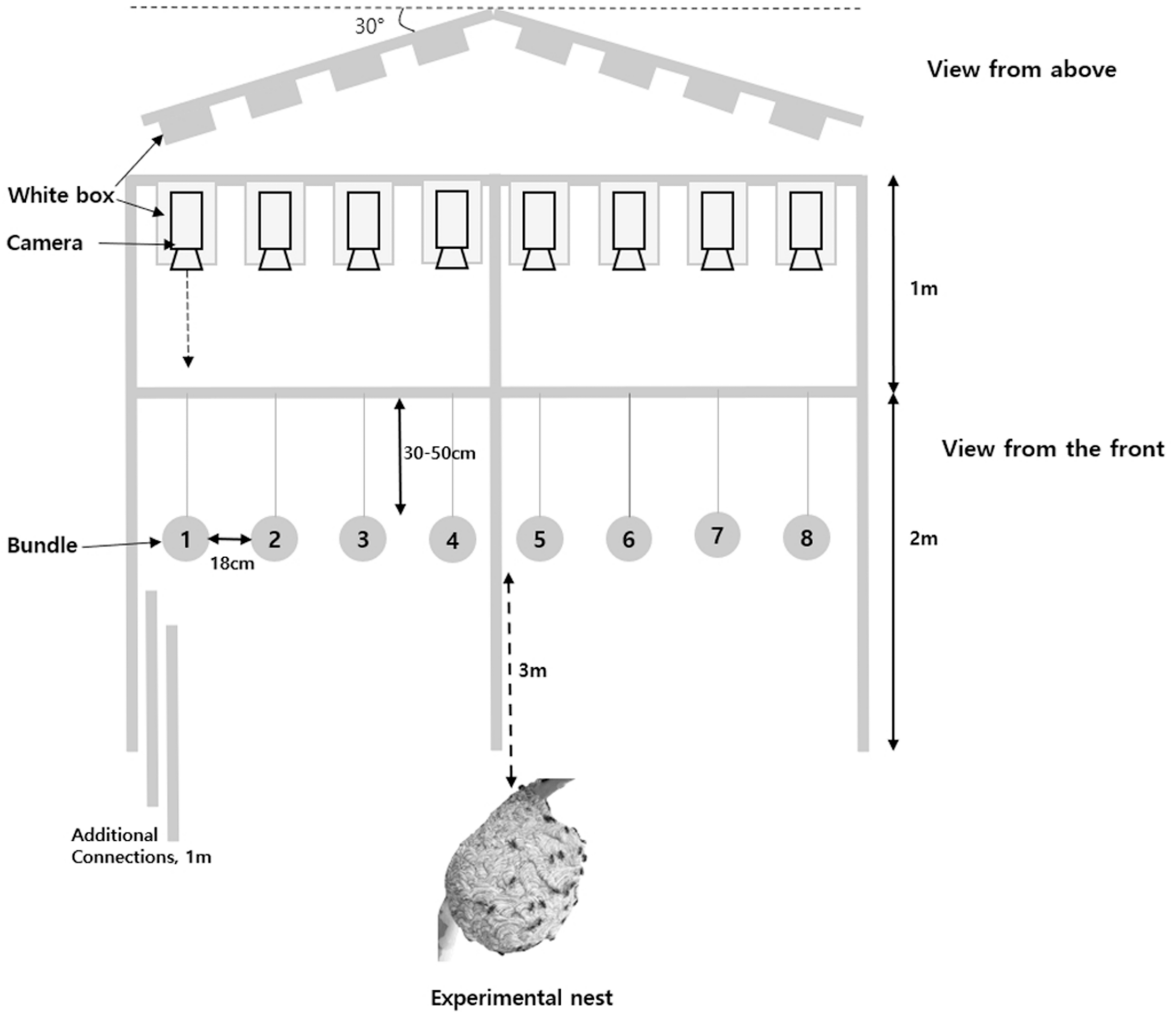
Experimental framework for the analysis of the defensive behavior *of V. velutina*.

The number of workers responding to the attack was counted and analyzed after taking a video of each target stimulus. To install targets (bundles) in front of the entrance of the nest, bundles were installed at equal intervals (18 cm) in the middle of an assembled white steel frame and one video camera (V 2000 Digital Camcorder, Sunwoo Tech. Corp., Goyangsi, Korea) per bundle was installed 1 m above the frame (Fig. 1). This device could accurately record the number of workers attacking a bundle without a blind spot. In addition, this frame was made at an angle of about 30° to maintain the same distance from the entrance of the nest to bundles located in the middle from both edges. The workers were counted using captured video files slowed by 8–16 times and only workers in contact with the bundle were defined as using defensive behavior. In addition, the measurements of the duration and final time of the defensive behavior were observed from the first defensive behavior of the workers to completion of the last defensive behavior. When workers attacked, the camera could be attacked, so the camera was placed in a white box and installed in a frame to minimize confusion with the target. This frame was used to adjust the height according to the nest and was installed ~3 m from the entrance of the nest.

### Color

The defensive behavior experiment relating to color was conducted using eight bundles of differently colored yarn, the Hex and RGB codes of which are as follows: black (Hex: #171716; RGB: 23, 23, 22), brown (Hex: #473010; RGB: 71, 48, 16), yellow (Hex: #eded21; RGB: 237, 237, 33), green (Hex: #5bf59d; RGB: 91, 245, 157), orange (Hex: #e38930; RGB: 227, 137, 48), grey (Hex: #acadac; RGB: 172, 173, 172), red (Hex: #ab1e0c; RGB: 171, 30, 12) and white (Hex: #faf5f5; RGB: 250, 245, 245). These bundles, each of which was 15 cm in length, 15 cm in height and 7 cm wide and consisted of 30% wool and 70% acrylic fibers. We conducted two experiments to understand the defensive behavior of workers against different colors and against one color: an eight-color test (ECT) in which all eight bundles of different colors were hung on the test frame, and a single-color test (SCT) in which one bundle of each color was hung at a time. In these two experiments, the defensive behavior of the workers against the targets was filmed on video from the first stimulus until the last attack was completed, and the number of workers, and the final time of defensive behavior was measured.

Therefore, the experiment was conducted at each of nine nests, and a total of 144 bundles were tested. The bundles were replaced for each experiment to avoid confounding, as an alarm pheromone is sprayed on the bundle (Cheng et al., 2017) when the workers attack. The order of color bundles on the test frame was randomly arranged for each experiment (Table 2). First, an ECT was performed, followed by a SCT. In the SCT, if the location of the bundle was fixed, there may have been a confounding effect of learning on the location, so the experiment was conducted by changing the location of the bundle points 3 and 6 while changing colors at random.

**Table 2:** Order of bundles of eight colors and bundles of single colors hung on the experimental frame for analysis of the defensive behavior of *V. velutina*.

Nest no.	Bundle	The points where the bundles are held in the test frame							
		1	2	3	4	5	6	7	8
1	ECT	Brown	Green	Black	Grey	Yellow	White	Orange	Red
	SCT	Green	Yellow	Brown	White	Black	Grey	Red	Orange
2	ECT	Yellow	Grey	Brown	Green	Orange	Black	Red	White
	SCT	Green	Black	White	Brown	Red	Grey	Orange	Yellow
3	ECT	Grey	Orange	Green	Red	Black	Yellow	Brown	White
	SCT	Red	Black	Brown	Orange	Green	White	Yellow	Grey
4	ECT	White	Brown	Red	Black	Grey	Yellow	Orange	Green
	SCT	Grey	Green	White	Black	Yellow	Brown	Red	Orange
5	ECT	Black	Red	White	Yellow	Brown	Orange	Green	Grey
	SCT	Orange	Yellow	Grey	Green	Brown	Red	Black	White
6	ECT	Orange	Yellow	White	Brown	Green	Grey	Black	Red
	SCT	Black	Red	Brown	Orange	Yellow	Green	White	Grey
7	ECT	White	Grey	Black	Green	Red	Orange	Brown	Yellow
	SCT	Grey	Green	Yellow	Black	White	Brown	Orange	Red
8	ECT	Green	Brown	Orange	Yellow	White	Grey	Red	Black
	SCT	Yellow	Orange	Brown	Red	Grey	Green	Black	White
9	ECT	Brown	Yellow	Green	Grey	Orange	Black	White	Red
	SCT	Orange	Green	Grey	White	Yellow	Brown	Red	Black

**Note:**

ECT, eight-color test; SCT, single-color test.

In addition, if the nest is continuously stimulated, workers may habituate to such disturbances and defense behavior may weaken. Therefore, the interval between experiments was set to 30 min to minimize the attenuation of defensive behavior.

### Hair

To assess defensive behavior against hair, we made bundles (same size as previous experimental bundle) of black, faux mink, hair fabric with a fiber length of 25 mm (Minkfur-black/CB8424, Wondannara Inc., Daegu, Republic of Korea). For hairless bundles, all the hairs of the same fabric were cut to less than 1 mm using a hair clipper. There were also nine bundles and they were tested with a total of 18 bundles. These bundles were tested for number of workers, duration and final time of defensive behavior under the same conditions as the previous experiment, and the test was carried out by alternately hanging hair at points 3 and 6 of the test frames. The duration was measured at 30 s intervals until the last worker fell from the bundle.

We also compared the defense responses of workers when presented simultaneously with color and hair stimuli. We made nine bundles of green hair (same conditions as the previous black hair bundle except that it was green; Minkfur-green/TG8710), and nine, black, hairless bundles.

These experiments were conducted 30 min after the previous color experiment was completed, and the experiments were also conducted within 30 min of the previous experiment.

### Noise

In order to determine the defensive behavior in response to auditory stimuli, we counted the number of workers that responded to sounds broadcast from a portable wireless speaker (LG XBOOM Go PK7, white color, LG Electronics, Seoul). After connecting the speaker to a 50 cm stick, we slowly and carefully approached the nest entrance at a distance of 30 cm so that the workers did not react to the speaker object. As a stimulus, we used an increasing volume of music between 40 and 90 dB, and a range of quiet and loud human voices (Everyday sounds associated with humans such as conversations, laughter and music are typically produced at volumes of approximately 60 decibels (100–250 Hz) (Re et al., 2012)). Given that at 90 dB, the sound waves from the speaker could physically stimulate the nest via vibration, and that the worker may respond to this stimulus, we did not exceed this limit. The volume of the projected noise was measured using a sound level meter (Model: MT-4618; Proskit, Taiwan).

### Statistical analysis

We analyzed the defensive behavior of *V. velutina* with respect to number of workers and final time against the eight color, and single-color bundles using a one-way analysis of variance in combination with a *post-hoc* Tukey’s honestly significant difference test. In addition, an independent *t*-test was performed to verify the comparisons between the number of workers and final time against black hairy and hairless bundles, and green hair and black hairless bundles. All analyses were performed using IBM SPSS Statistical software version 23.0 (IBM, Chicago, IL, USA).

## Results

### Color

**Figure 2:**
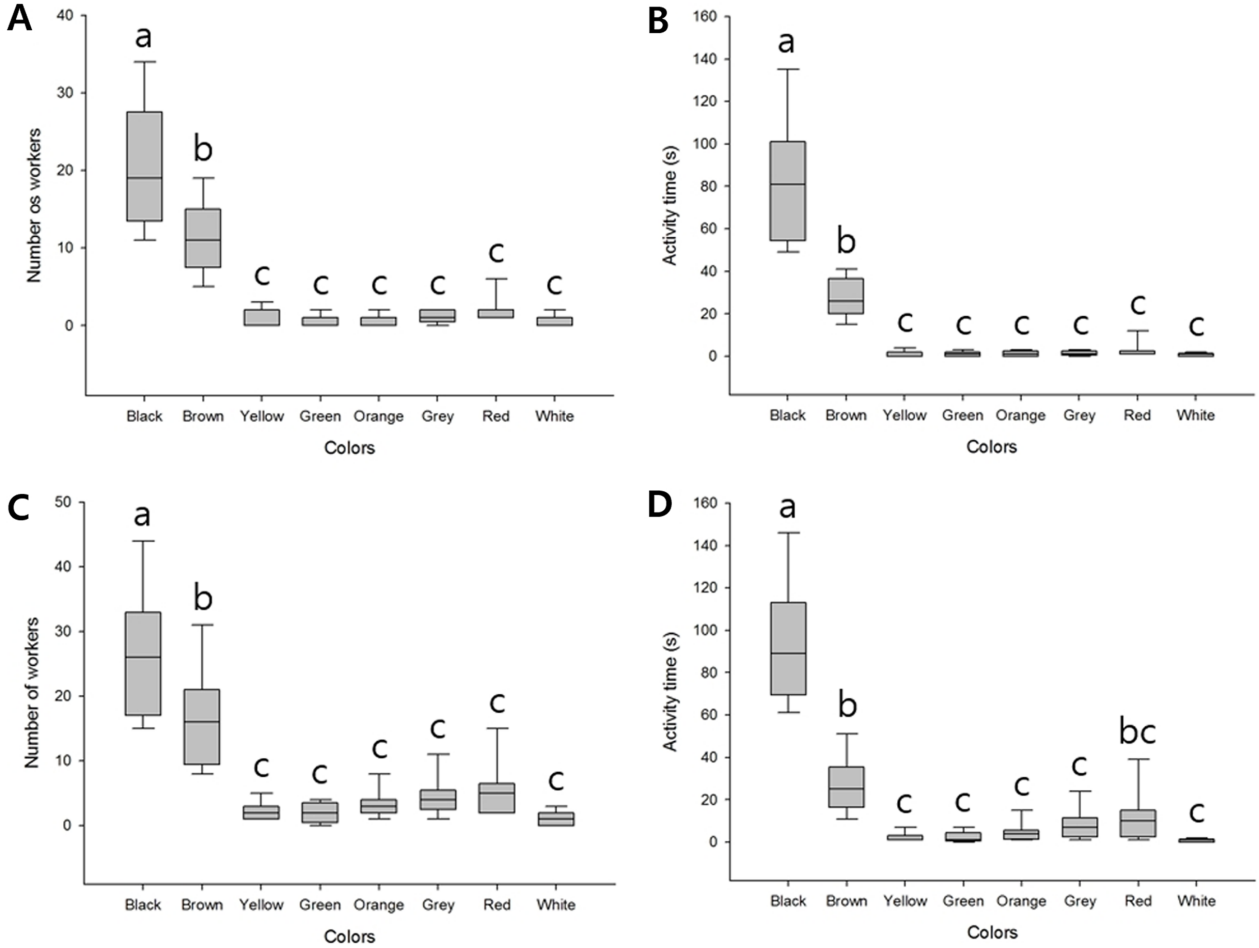
Defensive behavior of *V. velutina* workers in response to bundles of different colors. (A) The number of workers taking defensive behavior against eight colored bundles (black, brown, yellow, green, orange, grey, red and white), *F*_(7, 64)_ = 43.64, *P* < 0.001, (B) duration of the defensive attack of workers against the eight-color bundle, *F*_(7, 64)_ = 64.99, *P* < 0.001, (C) The number of workers taking defensive behavior against a single-color bundle, *F*_(7, 64)_ = 31.99, *P* < 0.001, (D) duration of the defensive attack of workers against a single-color bundle, *F*_(7, 64)_ = 63.25, *P* < 0.001.

We found that when presented with bundles of eight different colors, the highest number of workers responded to the black bundle (average 20.56 ± 7.89), followed by the brown bundle (average 11.11 ± 4.51); the difference was significant (*F*_(7, 64)_ = 43.64, *P* < 0.001). In contrast, a few workers appeared to show defensive behavior against the remaining six colors, and we detected no significant differences (Fig. 2A; Table S1). The final time of workers defending against these bundles was also longer for black (average 82.11 ± 28.53) and brown (average 27.44 ± 9.00) than the remaining six colors (*F*_(7, 64)_ = 64.99, *P* < 0.001). The remaining six colored bundles were of minor significance (Fig. 2B; Table S2).

When the different colored bundles were presented individually, since there was only one target, there were more workers attacking than when there were multiple color bundles. However, the tendency of defensive behavior was the same. Black (average 26.33 ± 9.57) and brown (average 16.22 ± 7.32) bundles elicited higher responses (*F*_(7, 64)_ = 31.99, *P* < 0.001, Fig. 2C; Table S3) and the final time was similar (*F*_(7, 64)_ = 63.25, *P* < 0.001, Fig. 2D; Table S4). In addition, of the minor bundles, red was slightly higher, but there was no statistical significance except for individual color attack time (Fig. 2D).

### Hair

**Figure 3:**
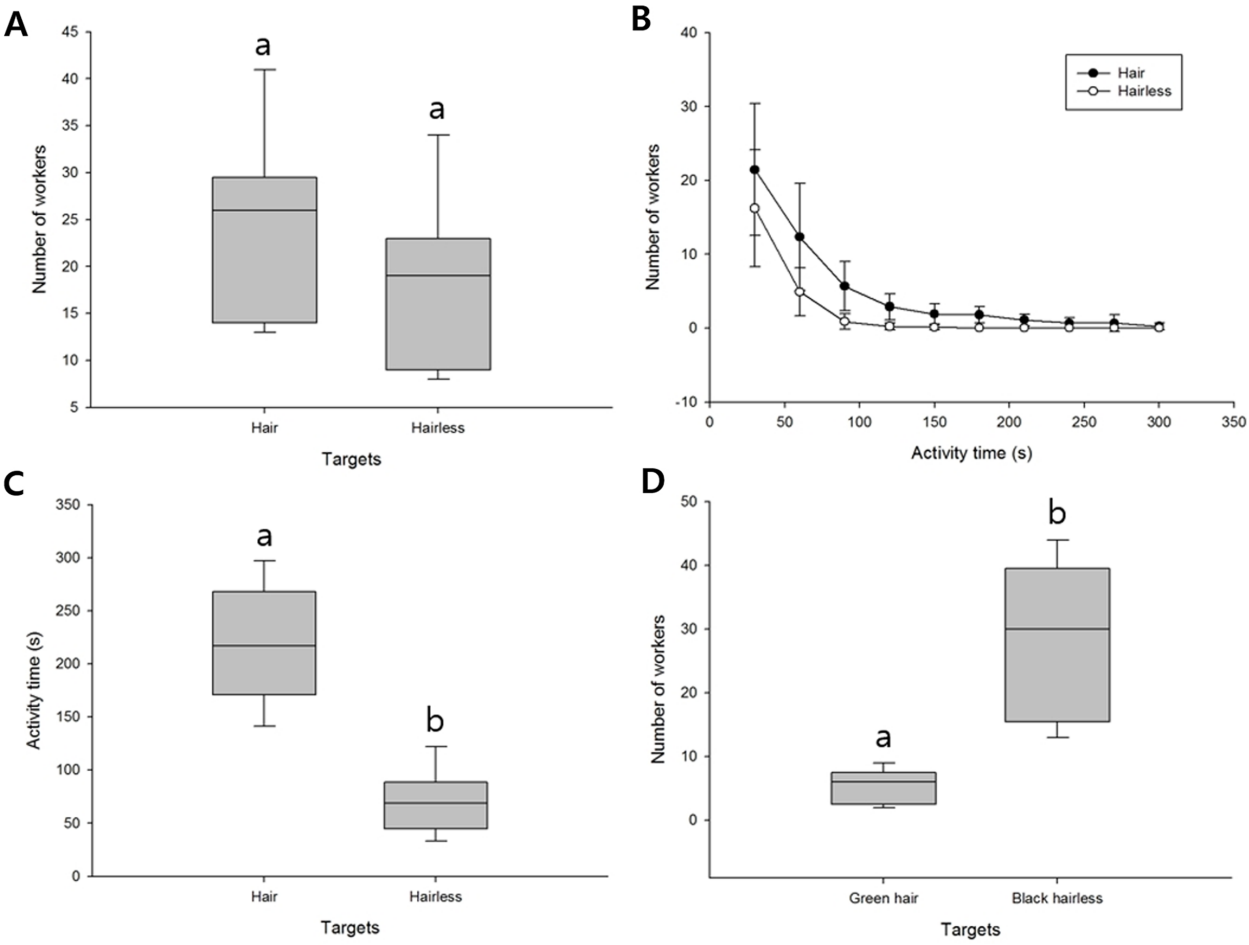
Defensive behavior of *V. velutina* workers in response to hair and color stimuli. (A) Comparison between hair and hairless black bundles, *t*_(16)_ = 1.412, *P* = 0.177, (B) duration of the defensive response against hair and hairless black bundles; error bars indicate standard deviation, (C) duration of the defensive response against hair and hairless black bundles, *t*_(16)_ = 7.292, *P* < 0.001, (D) comparison between green hair and black hairless stimuli, *t*_(16)_ = −5.653, *P* < 0.001.

When presented with black hairy and hairless bundles, the number of workers showing defensive behavior against the black-haired bundle was on an average 23.67 ± 9.31 and average number of the black hairless bundle was 17.78 ± 8.75. The black hairy bundle was slightly higher, but the difference was not significant (*t*_(16)_ = 1.412, *P* = 0.177, Fig. 3A; Table S5).

An average of 21.44 ± 8.93 workers showed defensive behavior against the black-haired bundle within 30 s, and the number of responding workers decreased to an average of 12.33 ± 7.24 at 60 s. Thereafter, the number declined to 10 workers, and the remaining workers remained for about 300 s before returning to the nest (Fig. 3B; Table S6). The black hairless bundle drew an initial response from an average of 16.22 ± 7.90 workers, which decreased to fewer than 10 workers after 30 s (average 4.88 ± 3.25), and no workers remained after 150 s (Fig. 3B; Table S7). Therefore, the final attack time against the targets was 218.44 ± 54.02 s against the hair bundle (Table S6), and 70.44 ± 28.09 s against the hairless bundle (Table S7). The defensive behavior against the hair lasted much longer (*t*_(16)_ = 7.292, *P* < 0.001, Fig. 3C). There was no significant difference in the number of workers attacking the hairy bundles, but they were attacked for longer.

When presented with green hair and a hairless black bundle, the hairless black bundle (average 28.11 ± 11.81) drew a higher defensive response than green hair (average 5.33 ± 2.55), thereby indicating that the workers were more responsive to color than to hair texture, per se (*t*_(16)_ = −5.653, *P* < 0.001, Fig 3D; Table S8).

### Noise

We found that workers showed no response to sound when exposed to music played at a volume between 40 and 90 dB.

## Discussion

### Color

Insect visual acuity plays an important role in searching and host awareness, finding mates, seeking shelter and locating foraging sites and food (D’adamo & Lozada, 2003; McNeill et al., 2016; Kafle, Gonzales & Neupane, 2019). Hymenoptera members are characterized by trichromatic color vision, which is based on three types of photoreceptors that are maximally sensitive in the UV, blue and green wavelengths (Peitsch et al., 1992; Briscoe & Chittka, 2001). In bees, these wavelengths are used when visiting flowers or performing other activities outside the nest, and social wasps respond to visual cues at similar wavelengths (Peitsch et al., 1992). Social wasps are highly attracted to green traps (Sharp & James, 1979; Jung, Kang & Kim, 2007). Primary colors are characteristic of the local environment of wasp nests, such as forest vegetation, where social wasps tend to be active, and the social wasps are not particularly alert. Accordingly, in the present study, we observed that hornet workers tended to show minimal responses to primary and bright colors.

In contrast, darker shades and blacks or browns, which are not commonly encountered in the surrounding environment, serve to alert wasps to a potential threat, particularly with respect to the mammalian and avian predators of wasps that are typically black, grey, or brown (Choi, Woo & Choi, 2015; Macià et al., 2019). In many cases, the plumage or fur of predators is dark, and thus it appears that social wasps have become evolutionarily adapted to show a heightened defensive behavior against dark colors (Nadolski, 2013). Indeed, *V. mandarinia* (Kim & Jung, 2019) and *V. crabro* (Nadolski, 2013) show similar responses to dark colors. However, consistent with the findings of the present study with respect to the response to red, Nadolski (2013) observed a significantly high defensive response against orange. Although such responses are not immediately explicable, in many predatory animals and insects, the red color of the opponent represents a warning and is an object of avoidance (Sillén-Tullberg, 1985; Roper, 1990). Therefore, it seems that defensive behavior is slightly higher because the object can be recognized more clearly against other natural colors. If presented with multiple colors, the workers will concentrate their attacks on black and brown materials, but even a single primary color elicits weak defensive behavior.

### Hair

Workers showed, on average, a strong defensive response against hair, but the difference was not significant, so the increase in aggression to hair was not proven. However, it was clearly shown that the duration of defensive behavior was much longer in the presence of hair. Hair and feathers are characteristic features of the mammalian and avian natural enemies of wasps and were expected to induce more defensive behavior, but in reality, most defensive behavior seemed to be triggered by color. Nevertheless, hair facilitates successive bouts of stinging, as it provides a physical support to which wasps can cling using their claws when attacking. Consequently, when attacking humans, if their hair is dark, wasps can often lengthen their attacks on the head region, thereby causing potential serious injury or death (Vetter, Visscher & Camazine, 1999; O’Neil, Mack & Gilchrist, 2007; Ito, Imafuku & Nakayama, 2012; Xie et al., 2013; Chowdhury et al., 2014).

Moreover, when wasps were simultaneously presented with green hair and a hairless black bundle to determine whether it was the color of the hair that was the primary stimulus for defensive behavior, the intensity of the defensive response was determined to a greater extent by the color more than the hair texture. Accordingly, we observed a particularly strong and prolonged attack when the wasps were presented with a black hair stimulus.

### Noise

Hornet workers exposed to sounds of up to 90 decibels failed to produce any defensive response. Given that ultrasonic pest repellent devices for insects are based on ultrasonic frequencies ranging from 30 to 50 kHz (Yturralde & Hofstetter, 2012; Abdulrahman, Amoo & Muhammad, 2019), it would seem that reducing human-associated noise in the vicinity of hornet nests would be ineffective in preventing a hornet attack. However, if sounds are sufficiently loud, the physical vibrations transmitted by sound waves could be detected by wasps within a nest, and this may be sufficient to provoke the workers’ defensive behavior.

### Possibility of defensive behavior against odor stimuli

Numerous insects have been found to respond to food, hosts and natural enemies by detecting characteristic chemicals, such as those comprising odors (Day & Jeanne, 2001; Jander, 1998; Jones, 2013; Webster & Carde, 2016). Flowering plants produce fragrant or honey-sweet odors that attract pollinating bees and flies (Ollerton, Winfree & Tarrant, 2011), whereas mosquitoes (Gillies, 1980; Healy & Copland, 1995), ticks (McMahon & Guerin, 2002), tsetse flies (Voskamp, Everaarts & Den Otter, 1999) and reduviid bugs (Barrozo & Lazzari, 2006) often detect CO_2_ in the body odors or exhaled breath of potential host animals. In addition to being attracted by such olfactory stimuli, when insects detect unfamiliar chemicals in their surroundings, they may sense potential danger and accordingly take evasive or defensive action (Boeve, Honraet & Rossel, 2014; Khan et al., 2019; Maia & Moore, 2011).

Some wasps are similarly attracted to substances in human body odors, and may exhibit certain aggressive behavior if they happen to come in contact with humans (Akre & Davis, 1978), In particular, they appear to be most sensitive to changes in CO_2_ concentration (Jones, 2013).

When an intruder approaches the nest of social insects, such as that of honeybees and bumblebees, there is an increase in local CO_2_ concentration and the transmission of other multi-modal sensory stimuli, such as thermal gradients and various compounds in exhaled breath. Workers detecting these stimuli can take evasive or defensive action, as necessary (Jones, 2013).

Although the response of wasps to odors was not specifically examined in the present study, the defensive behavior of wasps could readily be assessed using human-produced odors and CO_2_, and such studies should be conducted in the future.

### Proposed countermeasures against social wasp attacks

In Korea, the incidence of social wasp stings is most frequent in the summer and autumn months when wasp nests and populations are at their largest and annual human outdoor activity tends to be at its highest. The risk of such encounters is notably heightened during *Chuseok* (Korean thanksgiving day), when most Koreans visit the graves of their ancestors and mow the surrounding grass. During the course of these activities, many individuals inadvertently disturb the nests of certain *Vespa* species, which are often found in the vicinity of forest tomb sites. This disturbance often results in an attack by swarms of wasps, causing serious injury and, in severe cases, even death (Choi, Kim & Kwon, 2019). Moreover, as indicated by observations made in the present study, the larger the colony, the larger is the number of workers that tend to attack. Accordingly, the severity of hornet attacks is likely to be greatest when large nests are disturbed in early autumn. In particular, *V. velutina*, which is increasing in density in urban areas, often stings people in residential areas and urban parks as some of their nests occur within direct human contact (Choi, Kim & Lee, 2012; Choi, Martin & Lee, 2012).

In order to minimize the likelihood of *V. velutina* attacks, it would be desirable to establish effective countermeasures based on creditable, scientific information. On the basis of the findings of the present study, we can make the following recommendations in this regard. *Vespa velutina* attacks can be mitigated to a certain extent by wearing brightly colored or white clothing with a smooth texture, which would be particularly applicable to those whose work is likely to bring them in close contact with wasps, such as beekeepers and wasp hunters, as well as those weeding graves, and accidentally encountering nests in urban green areas. Moreover, given that most Asians have dark brown or black hair, it is also very important to wear a light-colored hat, as most wasps direct their attacks against the head area of humans. Furthermore, some actions should be avoided if close to a nest, as should any provocative behavior that might be conducive to triggering a wasp attack (Nadolski, 2013; Kim & Jung, 2019).

Importantly, if a nest is detected, attempts should not be made to remove it independently, but instead contact an emergency rescue team or an expert who will be able to take the appropriate measures. By following these precautionary rules, it should at least be possible to minimize the likelihood of wasp attacks.

## Conclusions

Being stung by this species is becoming more frequent as the spread and density of *V. velutina* increases. However, behavioral information is needed to respond appropriately to such sting accidents because damage continues to occur owing to incorrect or insufficient information. As a result of analyzing various defense behaviors of *V. velutina*, it was observed that defense actions are induced by characteristics such as black, dark brown, dark hair, hair and physical stimuli. Therefore, places in which humans can contact wasps or nests, such as when climbing in the forest, weeding, or working in urban green areas, it is best to wear bright clothes with a smooth texture. It is difficult to recognize the nests in the grass but it is advisable not to approach nests as much as possible. If a person uncovers a nest or has been attacked, they should run away from the nest and go to the hospital as soon as possible. The nest can be dealt with by a wasp specialist.

## Supplemental Information

10.7717/peerj.11249/supp-1 Supplemental Information 1 Raw data of *V. velutina*’s defensive behavior experiment. Number of workers

## Additional Information and Declarations

### Field Study Permissions

The following information was supplied relating to field study approvals (i.e., approving body and any reference numbers): Gyeongbuk National University and Gayasan National Park were given verbal permission during the field experiment. Kyungpook National University’s nest was tested by Mr. Eunmo Kang of the Operation Support Team, Division of General Administration of Kyungpook National University requesting and controlling the nest in the campus. In the case of national parks, Dr. Jong-cheol Jeong of the National Park Research Institute was the assistant of the experiment, and the experiment was conducted under the permission of the national park.

### Data Availability

The following information was supplied regarding data availability: The raw measurements are available in the Supplemental File.
